# Liver Biopsy and FibroScan to Detect Early Histopathological Changes in Chronic HBV Patients Not Candidate for Treatment

**DOI:** 10.14740/gr597w

**Published:** 2014-05-02

**Authors:** Sahar Maklad, Gamal Esmat, Ehsan Hassan, Mohamed Attalah, Alaa Abou Zeid

**Affiliations:** a Hepatology and Gastroenterology, National Hepatology and Tropical Medicine Research Institute, Kasr Al Ainy Street, Fum El Khaleeg, Cairo, Egypt; b Tropical Medicine and Hepatology, Faculty of Medicine, Cairo University, Tagamoe Khamis, New Cairo, Egypt; c Pathology Department, National Hepatology and Tropical Medicine Research Institute, Alharam, Giza, Egypt; d Public Health, Faculty of Medicine, Cairo University, 1 Lebanon Square, Mohandesin, 12411, Cairo, Egypt

**Keywords:** HBV, Egypt, Liver biopsy, FibroScan

## Abstract

**Background:**

We aimed at evaluating liver biopsy and FibroScan (FS) to assess early histopathological changes among chronic hepatitis B virus (HBV) patients not candidates for treatment.

**Methods:**

One hundred thirty-five chronic hepatitis B naive patients were followed up twice weekly at National Hepatology and Tropical Medicine Research Institute. All patients were not candidates for treatment according to both Egyptian and international guidelines. Pre-enrollment assessment was performed through biochemical, serological and quantitative HBV DNA testing. Liver biopsy was performed to 59 patients based on the guidelines while FS was performed to patients who were not candidates for liver biopsy (102 patients). Twenty-six patients performed both liver biopsy and FS (isolated liver biopsy 33 patients and isolated FS 76 patients).

**Results:**

At the end of study period, liver biopsy group showed that majority of subjects had grade F1 fibrosis (61.0%). Only 13.6% were F3. FS showed that almost half (47.1%) of subjects had a grade of F0 and 21.6% with grade F1. Only 4.9% of subjects had fibrosis grades of F3 or F4. In each test, nearly two-thirds of patients had evidence of F0/F1 fibrosis and the remaining one-third had more marked fibrosis. The degree of fibrosis as detected by both liver biopsy and FS was directly related to alanine aminotransferase (ALT), aspartate aminotransferase (AST), S. albumin and prothrombin. Patients with advanced fibrosis had significantly higher ALT and AST, while their S. albumin and prothrombin were significantly lower than those with minimal fibrosis.

**Conclusion:**

FS study requires further validation in HBV but could be confidently used at the present time as a predictor for the degree of hepatic fibrosis in chronic HBV patients. Liver biopsy could be spared for cases that present with elevated liver functions and/or marked impairment of synthetic liver functions.

## Introduction

Worldwide, over 360 million people are chronically infected hepatitis B virus (HBV) [1]. Chronic HBV infection is associated with increased risk of developing cirrhosis, hepatic decompensation and hepatocellular carcinoma [2]. Approximately 10-20% of patients with chronic hepatitis B (CHB) infection have liver cirrhosis at first presentation, and an additional 20-30% of patients will eventually develop this condition and its complications within one or more decades [3]. Previous studies indicated an annual risk of developing hepatocellular carcinoma (HCC) of 1-6%, and a similar or higher risk of hepatic decompensation after the development of cirrhosis. Although antiviral treatment using nucleot(s)ide analogues (NUCs) suppresses HBV effectively, we still face many complications in spite of effective treatment [4].

Serum HBV DNA level, in addition to elevation of liver enzymes, is used as a criterion for antiviral treatment in patients with CHB [5]. Relationship between serum HBV DNA level and liver histology remains controversial. Most patients who were previously considered to have non-replicative infection have detectable serum HBV DNA and HBV replication persists throughout the course of chronic HBV infection [1]. Most of HBV treatment guidelines have reserved liver biopsy for cases that do not really meet clear cut-off values of treatment or where there is a discrepancy between high viral load (≥ 2,000 IU) and normal liver enzymes or vice versa. Most guidelines recommend treatment of chronic HBV for those with high HBV DNA ≥ 2,000 and persistently elevated liver enzymes. HBV-infected patients with alanine aminotransferase (ALT) values close to the upper limit of normal were found to have abnormal histology and can be at increased risk of mortality from liver disease especially those above the age of 40 years [6].

All other chronic HBV patients who do not meet those guidelines are not candidates for treatment, but only for follow-up. Many recent studies have stated that this group of patients may have a silent ongoing liver disease which may not really be apparent in HBV DNA levels or elevated enzymes, therefore are not offered any treatment and may pass into complications directly and discovered very late [7]. Hepatic fibrosis is a consequence of all chronic liver diseases [8]. The methods of diagnosing hepatic fibrosis have evolved over the last few years. Liver biopsy has been considered until now as the gold standard for evaluating hepatic fibrosis [9]. However, it may be considered invasive and expensive procedure, and its accuracy is reduced due to sampling errors, inadequate specimens and inter- and intra-observer variations in histological interpretation [10].

Needle biopsy of the liver (NBL) is also clearly unsuitable for monitoring disease progression, especially in untreated patients. Patients with active HBV replication do not always have NBL, and many therefore remain untreated [11].

An alternative method for evaluating liver fibrosis is therefore needed to optimize patient management in resource-limited countries. This would also simplify access to treatment.

Transient elastography (FibroScan (FS), Echosens, France) is a reproducible, non-invasive and rapid method for measuring liver stiffness [12]. It is based on sonographic measurement of the propagation velocity of an elastic wave induced by the device itself: the faster the wave, the stiffer the medium. Studies of patients with hepatitis C virus (HCV) infection have shown that wave velocity correlates with the degree of liver fibrosis, and that elastography can accurately detect both early fibrosis and advanced fibrosis [13].

However, few data are available on the use of this method in patients with hepatitis B, especially in Egypt.

## Methods

Between January 2012 and December 2013, a total of 135 chronic naive CHB patients regularly attending HBV specialized clinic at the National Hepatology and Tropical Medicine Research Institute (NHTMRI) on twice weekly basis were recruited. Patients were not candidates for HBV treatment according to Egyptian guidelines for HBV which state that inclusion criteria for treatment are: age ≥ 18 years, hepatitis B surface antigen (HBsAg) (+ve) for more than 6 months, HBV DNA ≥ 2,000 IU/mL, ALT elevation above upper limit of normal on two successive occasions within 3 - 6 months.

According to the Egyptian guidelines, liver biopsy is used to guide treatment decisions for patients who show: 1) HBV DNA ≥ 2,000 IU/mL with persistently normal ALT; 2) HBV DNA < 2,000 IU/mL with persistently elevated ALT; and 3) HBV DNA < 2,000 IU/mL with normal ALT and there is clinical evidence of liver disease or a family history of HCC. Treatment is recommended for those with A2 and/or F2 or more (Metavir score) (released on March 31, 2013, unpublished data).

They were only candidates for follow-up by regular investigations for liver profile and HBV DNA every 3 - 6 months decided on a case-by-case basis. Inclusion criteria for our patients were adult males and females above 18 years old, HBeAg positive or negative, HBV DNA level ≥ 2,000 IU/mL for liver biopsy group (group 1), any viral load for FS group (group 2) and normal or fluctuating liver enzymes.

Exclusion criteria included concomitant HCV infection, evidence of liver disease induced by other causes, pregnancy and age less than 18 years.

NHTMRI IRB approved our study and a written informed consent was taken from all subjects.

A structured and tested case report form (CRF) was developed and used to collect patients’ data. The clinical parameters in the CRF included present and past medical history, and biochemical parameters including ALT, aspartate aminotransferase (AST), albumin, total bilirubin, platelet count and prothrombin conc. Serological markers included HBsAg, hepatitis B surface antibody (HBsAb), HBeAg, HBeAb and HCV antibody (HCV Ab) and quantitative HBV DNA testing.

Patients used to come every 3 months for clinical examination and follow-up of liver profile, and every 6 months for HBV DNA quantitative, alpha-fetoprotein and abdominal ultrasound. Follow-up period of our patients was variable according to each case individually.

Liver biopsy was performed to the first group of patients (59 cases) according to Egyptian guidelines to assess the degree of liver fibrosis and necroinflammation before starting antiviral treatment in patients with viral load ≥ 2,000 IU with fluctuating or persistently normal liver enzymes.

FS study was performed to the other group of patients (102 cases) who have a viral load below 2,000 IU/mL with persistently normal or fluctuating level of enzymes and are not considered candidates for any treatment. Cases that performed isolated liver biopsy were 33 cases (24.4%) while cases who performed isolated FS were 76 case (56.3%). A third group of patients comprised of 26 patients performed both liver biopsy and FS.

Five cases were excluded due to failure of FS because of obesity specially that we were using a medium sized probe (the only one available in our institute) which did not work for those patients due to characteristic thoraco abdominal fat, and hence excluded from the study.

Data were entered in MS Excel 2010 sheet. Data cleaning was done and then the data were transformed to Statistical Package for the Social Sciences (SPSS) version 20.0 for statistical analysis. Frequency and percent were used to present qualitative variables and mean ± standard deviation was used to present quantitative data. The chi-squared test was used to estimate difference between groups regarding qualitative variables to highlight differences in different parameters based on liver biopsy and FS readings.

## Results

A total of 135 patients were included in our study. The age of our patients ranged 18 - 56 years old with a mean of 33 (Table 1).

**Table 1:** Description of Study Subjects in Relation to Age and Some Blood Parameters

	Mean	Std. deviation	Minimum	Maximum
Age	33.0	± 9.0	18.0	56.0
ALT	34.2	± 23.4	10.0	168.0
AST	31.0	± 15.7	8.0	124.0
Total bilirubin	0.9	± 1.5	0.3	17.0
Alpha-fetoprotein	4.2	± 3.4	0.3	23.0
S. albumin	5.8	± 0.49	2.0	5.2
Prothrombin	87.1	± 8.6	62.0	110.0
S. creatinine	0.88	± 0.23	0.4	2.1
Platelet	229.6	± 52.7	61.0	384.0
HBV DNA	8,262,839	± 36,755,837	52.0	308,000,000

Both genders were included in our study. Males were predominant in this study (84.4%). Most of the patients were HBeAg negative (91.9%), HBeAb positive (91.9%) and HCV Ab negative (94.8%) (Untabulated data).

Biochemical parameters of our patients revealed normal or slight fluctuations regarding liver enzymes. Alpha-fetoprotein was variable among patients with some patients having an alpha-fetoprotein level above 20. HBV DNA showed heterogeneous values ranging from 52 up to 308 million (Table 1).

Abdominal ultrasonography showed that study participants had hepatomegaly in 29.6%, bright liver in 28.1% and coarse liver in 18.5%. Almost one-fourth of patients are shown having normal liver (23.7%) (Table 2).

**Table 2:** Results of Abdominal Ultrasonography for Diagnosis of Liver Disease

Variable		Frequency	Percent
Abdominal ultrasound	Bright liver	38	28.1
	Coarse	25	18.5
	Hepatomegaly	40	29.6
	Normal	32	23.7

According to Metavir score, liver biopsy showed that majority of subjects had a grade F1 fibrosis (61.0%), 20.3 % of our patients were F2, 3.6% were F3 and only 5% were F0. FS showed that almost half (47.1%) of subjects had a grade of F0 and 21.6% with grade F1. Only 4.9% of subjects had fibrosis grades of F3 or F4 (Table 3).

**Table 3:** Metavir Classification of Results of Liver Biopsy in the Study Patients Corresponding FibroScan Results Correlated With Metavir Scoring System

Variable		Number	Percent
Liver biopsy*	F0	3	5.1
	F1	36	61.0
	F2	12	20.3
	F3	8	13.6
FibroScan*	F0	48	47.1
	F1	22	21.6
	F2	27	26.5
	F3	3	2.9
	F4	2	2.0

* Cases that performed isolated liver biopsy were 33 cases (24.4%) while cases who performed isolated FibroScan were 76 case (56.3%). A third group of patients comprised of 26 patients performed both liver biopsy and FibroScan.

For further clarification of our results, we tried to classify our patients into minimal fibrosis (F0-F1) and advanced fibrosis groups (F2-F3). Results of liver biopsy showed that almost two-thirds of patients have minimal fibrosis while 33.9% have advanced fibrosis. Similarly, FS showed that 68.6% of subject had minimal fibrosis versus 31.4% with advanced fibrosis (Table 4).

**Table 4:** FibroScan As a Predictor for Liver Fibrosis

Variable	FibroScan	Mean	Std. deviation	P value
Age	Minimal fibrosis	32.7	8.3	0.005
	Advanced fibrosis	39.7	9.4	
ALT	Minimal fibrosis	27.1	9.5	< 0.001
	Advanced fibrosis	49.3	24.4	
AST	Minimal fibrosis	27.3	12.1	< 0.001
	Advanced fibrosis	42.3	14.0	
A fetoprotein	Minimal fibrosis	3.6	2.2	< 0.001
	Advanced fibrosis	6.2	2.6	
S. albumin	Minimal fibrosis	4.3	0.44	0.007
	Advanced fibrosis	4.1	0.56	
Prothrombin	Minimal fibrosis	90.5	6.5	< 0.001
	Advanced fibrosis	79.1	8.2	
S. creatinine	Minimal fibrosis	0.86	0.2	0.015
	Advanced fibrosis	0.98	0.3	
Total bilirubin	Minimal fibrosis	0.7	0.2	0.820
	Advanced fibrosis	0.7	0.3	
Platelets	Minimal fibrosis	240.3	42.8	0.053
	Advanced fibrosis	216.6	45.6	
HBV DNA/IUML	Minimal fibrosis	10,543,144	39,095,338	0.240
	Advanced fibrosis	214,306	940,519	

The degree of fibrosis as detected by liver biopsy was found to be directly related to ALT, AST, S. albumin and prothrombin conc. Patients with advanced fibrosis had significantly higher ALT and AST, while their S. albumin and prothrombin were significantly lower (Table 5).

**Table 5:** Liver Biopsy As a Predictor for the Degree of Liver Fibrosis

Variable	Liver biopsy	Mean	Std. deviation	P value
ALT	Minimal fibrosis	27.2	12.5	< 0.001
	Advanced fibrosis	47.4	35.9	
AST	Minimal fibrosis	26.4	9.1	< 0.001
	Advanced fibrosis	37.6	21.9	
S. albumin	Minimal fibrosis	4.3	0.47	0.002
	Advanced fibrosis	3.9	0.39	
Prothrombin	Minimal fibrosis	89.6	7.4	< 0.001
	Advanced fibrosis	82.4	8.8	

There was no statistical difference comparing different variables regarding age, sex, total bilirubin, alpha-fetoprotein, serum creatinine, count or HBV DNA between patients with minimal fibrosis versus those with advanced fibrosis as indicated by liver biopsy (untabulated data).

Regarding FS, patients with advanced fibrosis had higher age as compared to those with minimal fibrosis (P = 0.005). The levels of ALT, AST, alpha-fetoprotein and serum creatinine were significantly higher in group with advanced liver fibrosis while serum albumin and prothrombin were significantly lower in the same group as compared to those with minimal fibrosis. The level of fibrosis was not related to total bilirubin level, platelets counts and HBV DNA.

## Discussion

Despite advances in serologic testing and imaging techniques, liver biopsy remains to be the gold standard and definitive test to assess the prognosis in most forms of parenchymal liver disease. The purpose of liver biopsy among chronic HBV patients is to assess the degree of liver damage and to rule out other causes of liver disease. Liver histology can improve significantly in patients who have sustained response to antiviral therapy or spontaneous HBeAg seroconversion and can also worsen rapidly in patients who have recurrent exacerbations or reactivations of hepatitis [14].

However, most of HBV treatment guidelines have reserved liver biopsy for cases that do not really meet clear cut-off values of treatment or where there is a discrepancy between high viral load (≥ 2,000 IU) and normal liver enzymes or vice versa.

Ultrasonic transient elastography, or FS (Echosens, Paris, France), is a new approach for the noninvasive evaluation of hepatic fibrosis. FS has been shown to be an accurate predictor of histological fibrosis in patients with chronic hepatitis C (CHC) [15]. However, the experience with FS in patients with chronic HBV infection (exclusively) is less extensive. In these patients, liver stiffness measurement (LSM) values may be limited by elevated ALT, and the LSM cut-off values for each disease stage vary from study to study due to differences in hepatic fibrosis etiology and differences in populations. It is well known that the staging of liver fibrosis (using METAVIR score F = 2-3 and F = 4) is crucial for making therapeutic decisions and for assessing the prognosis of chronic HBV infection [16].

HBV infection depends on prior diagnosis of liver fibrosis. Thus, accurate assessment of fibrosis is a prerequisite for improving the management of HBV-infected patients in resource-limited countries.

Our study is not a comparative study between liver biopsy and FS. We aimed at evaluating the degree of fibrosis that may accompany the clinical condition of our patients who are under follow-up without treatment especially with the increasing number of cases with low viral load and normal or fluctuating levels of liver enzymes. By using either liver biopsy or FS without making a direct comparison between the results of both procedures, we tried to re-evaluate those cases. Hence, liver biopsy was performed to patients who are eligible for liver biopsy while FS was performed to other patients who were not eligible for liver biopsy. This explains the greater number of FS cases if compared to liver biopsy. FS has been shown to be an accurate predictor of histological fibrosis in patients with CHC [12, 17]. However, the experience with FS in patients with chronic HBV infection is less extensive especially in Egypt.

We divided our patients into two groups: the first for patients who were eligible for liver biopsy according to guidelines. More than 66% of our cases had a minimal fibrosis score (36 cases (61%) of F1 and three cases (5.1%) of F0). Cases with advanced fibrosis represented about 13.6% where biopsy result was F3. The degree of fibrosis as detected by liver biopsy was related to ALT, AST, S. albumin and prothrombin. Patients with advanced fibrosis had significantly higher ALT and AST, while their S. albumin and prothrombin were significantly lower. This result could guide our decisions regarding the choice of our patients who would benefit from liver biopsy and could be offered the chance of treatment in spite of not following the usual guidelines of treatment.

Similar results were observed by Wong et al in 2013 as they found that liver disease was observed in 34.4% and 61.8% of patients with normal ALT and mildly elevated ALT, respectively [18]. Patients with mildly elevated ALT levels had significantly more events, including liver disease, elevated AST, and moderate to severe inflammation and liver fibrosis, than patients with normal ALT (all P ≤ 0.005). A total of 107 patients (46.5%) had liver disease and 123 (53.5%) did not. PLT and ALT were significantly associated with liver disease (both P < 0.001). Patients with elevated ALT, lower platelet count and HBV DNA < 7 log10 copies/mL may have histologically significant changes associated with liver disease. However, in our study population, HBV DNA level was not found to be a dominant factor behind the presence of significant fibrosis. This could be partially because many cases in our study were young patients and many were belonging to immune tolerant group of patients who had a significantly high HBV DNA and normal enzymes and minimal fibrosis score.

FS (the second group, 102 cases) results showed that 48 (47.1%) cases had a fibrosis score of F0 and 22 cases (21.6%) had a fibrosis score of F1, that shows that 68% of cases who do not meet clear guidelines of liver biopsy still have no or minimal fibrosis and are safely considered to be candidates for follow-up rather than for treatment. In our study, we estimated the mean levels for FS as F0 = 4.6, F1 = 6.3, F2 = 8.7, F3 = 10.7, F4 = 51.6.

Obesity has been consistently reported as a factor limiting the success of LSM in Western studies [19]. In our study, five patients failed because of obesity specially that we were using a medium sized probe which did not work for those patients due to characteristic thoraco abdominal fat, and they were excluded from our study. Cases with advanced fibrosis represented 31.4% and their clinical findings showed that the levels of ALT, AST, alpha-fetoprotein and serum creatinine were significantly higher in group with advanced liver fibrosis while serum albumin and prothrombin were significantly lower in the same group as compared to those with minimal fibrosis. This points that FS could be a useful predictor of development of advanced in CHB patients showing histologically advanced liver fibrosis. Kim and colleagues in 2012 showed similar results as their study showed that LSM can be a useful predictor of liver co-morbid problems [20]. However, some studies pointed to the fact that higher levels of aminotransferases can influence the liver stiffness values obtained by means of transient elastography, so that LSMs have to be interpreted in a biochemical context, otherwise there is a risk of overestimating the severity of fibrosis. Also this is why LSMs are not performed in acute hepatitis or during ALT flares in HBV chronic hepatitis [21, 22].

### Conclusion

In conclusion, FS could be considered a reliable predictor of significant fibrosis and cirrhosis in chronic HBV patients for majority of patients. According to our study, liver biopsy could be spared to cases who show persistent elevation of enzymes and or derangement of synthetic liver function or as a confirmatory tool for cases who present with advanced FS results.

### Recommendations

Further large-scale studies are needed to assess and validate FS in chronic HBV patients. Performing liver biopsy for patients who show advanced FS results could be considered after ensuring prompt ethical considerations.
